# Induction of chondrogenesis of human placenta-derived mesenchymal stem cells via heparin-grafted human fibroblast derived matrix

**DOI:** 10.1186/s40824-018-0121-2

**Published:** 2018-05-09

**Authors:** Yong Kwan Noh, Ping Du, Avelino Dos Santos Da Costa, Kwideok Park

**Affiliations:** 1 Center for Biomaterials, Korea Institute of Science and Technology, Seoul, 02792 South Korea; 2 Department of Biotechnology, Korea University, Seoul, 02841 South Korea; 3 Division of Bio-Medical Science and Technology, KIST School, Korea University of Science and Technology (UST), Seoul, 02792 South Korea

**Keywords:** Chondrogenesis, Human fibroblast-derived extracellular matrix (hFDM), Human placenta-derived mesenchymal stem cells (hPMSCs), Transforming growth factor (TGF-β1)

## Abstract

**Background:**

Formation of mature and functional articular cartilage is still challenging in cartilage tissue engineering. This study investigates the potential of using heparin-grafted decellularized extracellular matrix (ECM) as a novel growth factor delivery platform towards human placenta-derived mesenchymal stem cells (hPMSCs) chondrogenic differentiation. Human fibroblast-derived extracellular matrix (hFDM) is naturally obtained from in vitro-cultured human lung fibroblasts via a mild decellularization process. hFDM was then conjugated with heparin via N-(3-Dimethylaminopropyl)-N′-ethylcarbodiimide hydrochloride (EDC) chemistry and subject to transforming growth factor (TGF)-β1 immobilization. Once heparin grafted-hFDM (hFDM-hep) and hPMSCs were co-embedded into collagen gel, they were examined for in vitro and in vivo chondrogenesis of hPMSCs for 4 weeks.

**Results:**

We identified heparin moieties on hFDM via toluidine blue O assay and Fourier transform infrared spectroscopy, respectively. We found out that collagen spheroids containing hFDM-hep and TGF-β1 exhibited a sustained release of growth factor for 28 days in vitro. Chondrogenesis of hPMSCs in vitro was supported by accumulated glycosaminoglycan (GAG) content and upregulated chondrogenic specific markers (collagen II, aggrecan, Sox9). Meanwhile, PKH26 - labeled hPMSCs incorporated collagen with either hFDM or hFDM-hep was pre-conditioned in a chondrogenic media for 3 days and subcutaneously implanted in the back of nude mice for 4 weeks. The implanted collagen spheroids containing both hPMSCs and hFDM-hep retained more viable hPMSCs and showed higher level of chondrogenic differentiation, based on immunostaining of collagen type II over collagen alone or Col/hFDM group. In addition, histological examination showed more positive signals of GAG via Safranin-O staining.

**Conclusion:**

TGF-β1-immobilized hFDM-hep can provide an appropriate microenvironment for chondrogenic differentiation of hPMSCs in 3D collagen spheroid.

**Electronic supplementary material:**

The online version of this article (10.1186/s40824-018-0121-2) contains supplementary material, which is available to authorized users.

## Background

Osteoarthritis (OA) is a degenerative joint disease characterized by the destruction of articular cartilage [1]. Homeostasis of normal cartilage in adults represents a delicate balance between degradation and synthesis of extracellular matrix (ECM) of the tissue to maintain the functional integrity of the joint [2]. A breakdown of this cartilage matrix leads to the development of fibrillation, fissures and ulceration, together with gradual disappearance of the full-thickness joint surface. Moreover some pathological changes caused by OA involve not only cartilage itself but also the synovial membrane and subchondral bone [3]. Current treatment options are microfracture, periosteal, perichodrial grafts and bone marrow stimulation. Unfortunately cartilage has a limited self-repair capacity so that complete recovery of articular cartilage is a major hurdle [4].

Tissue engineering has emerged as a thriving new field in medical sciences. It is interdisciplinary field in which the principles of engineering and life science are integrated to generate biological substitute for improvement, maintenance, or restoration of organ functions [5, 6]. Formation of mature and functional articular cartilage is still challenging in cartilage tissue engineering [7, 8]. Tissue engineering generally requires three dimensional (3D) scaffold for cells and bioactive molecules. A typical extracellular microenvironment, for instance, ECM is very important in cell adhesion, migration and differentiation [9]. During tissue development, ECM is dynamically remodeled in accordance with cellular function [10]. In this sense, ECM based tissue engineering strategies are used widely for the regeneration of heart, trachea, muscle, tendon with matrices derived from bladder [11]. As a useful technique in preparing ECM, decellularization of tissues or cells is supposed to remove cells and cellular debris while retaining the bioactive cues that reside in the ECM [12]. The applications of ECM have been a great interest in tissue engineering such as, ECM incorporated scaffold, hybrid ECM, and grafted ECM [13–15].

Numerous studies demonstrate that mesenchymal stem cells (MSCs) contain multipotent cells and they can differentiate into bone, fat, cartilage, muscle, and neurons when induced by appropriate biological cues [16, 17]. MSCs are isolated from bone marrow, peripheral blood, placenta, amniotic fluid, umbilical cord blood, adipose tissue, and may perhaps be isolated from other sources in which they are resident components [18, 19]. Among these sources, placenta is an attractive source to isolate MSCs with multi-lineage differentiation capacity [20]. Because the placenta tissues are discarded after birth, they can be effectively utilized for research as well as clinical application with much less ethical concern [21].

In this work, the objective is to investigate the chondrogenic potential of human fibroblast-derived extracellular matrix (hFDM) and its derivative, heparin-grafted hFDM (hFDM-hep) as a transforming growth factor (TGF)-β1 delivery carrier in the induction of chondrogenesis of human placenta-derived mesenchymal stem cells (hPMSCs). To do this, hFDM was conjugated with heparin via EDC chemistry and subject to TGF-β1 immobilization. Once hPMSCs and hFDM or hFDM-hep were mixed with collagen gel, they were examined for in vitro and in vivo chondrogenesis of hPMSCs for 4 weeks. We hypothesize that TGF-β1 tethered hFDM-hep may provide an appropriate microenvironment for chondrogenic differentiation of hPMSCs.

## Methods

### Cell culture

Human placenta MSCs were obtained with informed consent and approval of the institutional review board of the School of Medicine, Sungkyunkwan University. The culture medium was Dulbecco’s modified Eagle’s medium-low glucose (DMEM; Gibco, USA) with 10% fetal bovine serum (FBS; Gibco) and 100 U/mL penicillin and 100 μg/mL streptomycin (P/S). For cell passage, the culture dish were washed with phosphate-buffered saline (PBS; Gibco) and incubated with TrypLE™ Select (Invitrogen, 12604-013) for 5 min at 37 °C in incubator. Dissociated cell suspensions were removed, than pelleted by centrifugation (1000 rpm, 5 min). Once the supernatant was discarded, cells were resuspended in culture media.

### Preparation of human fibroblast-derived matrix (hFDM)

WI-38 human lung fibroblasts (ATCC, CCL-75) were cultured at the cell density of 2 × 10^4^ cells/cm^2^ on the tissue culture dish (100 mm diameter) for 7 days in the DMEM supplemented with 10% FBS and 1% P/S. Once confluent, cell-loaded culture dish was washed twice with PBS, incubated briefly in a detergent solution containing 0.15% Triton X-100 (AMRESCO, Inc., Dallas, USA) and 10 mM NH_4_OH (Sigma; St. Louis, MO, USA) at 37 °C, and then treated with 50 U/mL DNase I and 50 μg/mL RNase A (Invitrogen) for 1 h. After the decellularization process, ECMs were collected into centrifuge tubes and stored at 4 °C for future usage.

### Heparin grafting onto hFDM

hFDM was washed with PBS and saturated with 0.05 M 2-(Nmorpholino) ethanesulfonic acid hydrate (MES) buffer (pH = 5.5) (M2933, Sigma). 0.25% (*w*/*v*) heparin working solution is prepared by adding heparin sodium (Acros, 41121-0010) to a freshly prepared solution of 0.05 M N-(3-dimethylaminopropyl)-N′-ethylcarbodiimide hydrochloride) (EDC; E7750, Sigma) and 0.06 M N-hydroxysuccinimide (NHS, 130672, Sigma) in MES solution; the EDC/NHS/MES solution was vigorously mixed and left for 10 min before the use. Eventually 2 mL of heparin working solution was added to hFDM samples in a 6-well plate. They were incubated at room temperature overnight while in rotating state.

### Characterization of hFDM-hep

The hFDM, hFDM-hep, and heparin sodium powder were analyzed using an attenuated total reflection (ATR)-4100 FTIR spectrometer (JASCO, Tokyo, Japan). The absorption spectra ranges between 650 and 2000 cm^− 1^. The baseline was automatically corrected using a background scan obtained in the absence of sample. Heparin conjugated hFDM (hFDM-hep) was also observed via toluidine blue O staining. 0.005% toluidine blue O (Sigma, T3260) solution was prepared in 0.01 N hydrochloric acid with 0.2% (*w*/*v*) sodium chloride (NaCl). The hFDM-hep was reacted with 2 mL of 0.2% NaCl and 0.5 mL of toluidine blue O solution for 1 h under shaking condition. The appearance of purple color indicates the presence of heparin on the hFDM. For quantitative analysis of heparin, we examined toluidine blue O-reacted solutions at 630 nm via Multiskan microplate reader (Thermo Scientific, Rockford, IL).

### Preparation of TGF-β1 immobilized hFDM and release test

Once hFDM-hep was ready in 6-well plates, 100 ng of TGF-β1 in PBS (1 ml) was added and incubated for 4 h at room temperature under a gentle shaking to incorporate TGF-β1 onto hFDM-hep. To monitor TGF-β1 (Peprotech) level tethered on hFDM, hFDM-hep was visualized using anti-human TGF-β1 antibody (Peprotech. 500 M-66) and Alexa Fluor 488 goat anti-mouse IgG (Invitrogen, A11017) and observed using fluorescent microscope (Olympus, CKX-41). For TGF-β1 release study in vitro, collagen (control) with TGF-β1 (100 ng/ml), and collagen spheroids containing hFDM/ TGF-β1 or hFDM-hep/TGF-β1 were put in a 6-well plate with 1 ml of PBS at 37 °C. 1 ml of PBS was collected at specific time points (1, 3, 7, 10, 14, 21, and 28 day) and additional PBS (1 ml) was replenished. Those collected samples were stored at − 20 °C before analysis. The release kinetics of TGF-β1 was determined using quantikine ELISA kit (R&D Systems). The cumulative percentage of TGF-β1 release was determined by normalizing the cumulative release of TGF-β1 at each time point with the total release amount of TGF-β1 over the course of 28 days.

### Evaluation of hPMSCs viability and cell morphology

hPMSCs (P9) were cultured on tissue culture plate (TCP), hFDM, and hFDM-hep substrates, respectively After 24 h of culture, we used LIVE/DEAD® Viability/Cytotoxicity Kit (Invitrogen, 03224) to evaluate cell viability. Live or dead cells were visualized in green and red fluorescence, respectively using fluorescent microscope. Evaluation of cell proliferation was carried out at 24 and 72 h using cell counting kit-8 (CCK-8; Dojindo, Japan). Briefly, samples were added with 10% CCK-8 solution and incubated at 37 °C for 2 h. Each supernatant (200 μL) was transferred to a 96-well plate and the absorbance was measured at 450 nm using a Multiskan microplate reader.

In addition, cell morphology on gelatin, hFDM, and hFDM-hep was also analyzed at 3 and 24 h, respectively. They were fixed with 4% paraformaldehyde for 30 min, gently washed with PBS, and permeabilized with 0.2% Triton X-100 for 20 min, then blocked with 1% BSA for 1 h. Each sample was incubated with primary antibody against vinculin (Santa Cruz, SC-73614) in 1% BSA (1:300) overnight at 4 °C. Washed three times with PBS, they were incubated with Alexa-Fluor-488-conjugated goat anti-mouse IgG (Invitrogen, A11001) in 1% BSA (1:200) for 1 h at room temperature in the dark, followed by incubation with rhodamine phalloidin (Invitrogen, R415) for 30 min. After being rinsed three times with PBS, they were mounted and observed using fluorescent microscopy.

### Preparation of collagen spheroids and chondrogenic induction of hPMSCs

To make collagen spheroids, hPMSCs suspension aliquots were mixed with rat tail type Ι collagen (3 mg/mL) and hFDM or hFDM-hep in an ice-bath. Collagen droplets (20 μL) were pipetted into a 6-well culture dish under UV-irradiation. We used a parafilm to cover the bottom of each well to prevent the adhesion of spheroids to the substrates. Those collagen spheroids underwent gelation when incubated at 37 °C in humidified atmosphere with 5% CO_2_ for 30 min. The chondrogenic differentiation medium was prepared using DMEM supplemented with 2% FBS, 1% P/S, 100 nM dexamethasone, 100 μg/mL proline, 100 μg/mL pyruvate, 1% ITS+ premix (BD Bioscience; 6.25 μg/mL insulin, 6.25 μg/mL transferrin, 6.25 μg/mL selenium, 5.33 μg/mL linoleic acid, and 1.25 μg/mL bovine serum albumin (BSA), 50 μg/mL ascorbate-2-phosphate (Invitrogen), and 10 ng/mL TGF-β1. For in vitro assay, Col and Col/hFDM were cultivated in the condrogenic media but TGF-β1 (50 ng/ml) tethered Col/hFDM-hep was cultured without TGF-β1 supplementation during 4 weeks.

### Glycosaminoglycan (GAG) assay

The GAG contents after induction of chondrogenic differentiation were measured by quantifying the amount of sulfated GAG using 1,9-dimethylmethylene (DMB) blue dye binding assay. DNA content of each sample was evaluated by using Quant-iT Picogreen dsDNA Assay kit (Invitrogen, Molecular Probes). The GAG content of each sample was normalized to that of DNA content.

### Quantitative real time polymerase chain reaction (Q-PCR)

Q-PCR was carried out to determine the gene expression level of chondrogenic markers. Total RNA was isolated from the samples (*n* = 3, each group) using TRIzol RNA Isolation Reagents (Invitrogen). The synthesis of first-strand cDNA was obtained from a solution of RNA extracts, primers and reverse transcription (RT) reaction mixture. The reaction product (1 μL) was then subject to the polymerase chain reaction (PCR) using Maxime PCR PreMix (Intron). PCR was performed via Applied Biosystems 7300 Real-Time PCR system using Taqman primers and probes. The relative gene expression was calculated using ΔΔC_T_ method, where each sample was internally normalized to glyceraldehyde-3-phosphate dehydrogenase (GAPDH).

Chondrogenic markers tested in this study are SRY-box containing gene 9 (SOX 9), type II collagen (Col II), aggrecan, and type Ι collagen (Col Ι). Target genes and their primers are as follows: SOX 9: AAAGGCAAGCAAAGGAGATG (forward) and TGGTGTTCTGAGAGGCACAG (reverse); Col II: AAGGCTCCCAGAACATCACC (forward) and ATCCTTCAGGGCAGTGTACG (reverse); Aggrecan: TCTGTAACCCAGGCTCCAAC (forward) and TGGAGTACCTGGTGGCTCTC (reverse); Col Ι: CTGGATGCCATCAAAGTCTTC (forward) and AATCCATCGGTCATGCTCTC (reverse); and control: GAPDH: GGGCTCTCCAGAACATCATC (forward) and TTCTAGACGGCAGGTCAGGT (reverse). The raw data were first normalized to GAPDH, and then normalized to collagen samples (0 day). The results were reported as a fold change relative to that of collagen (0 day).

### Subcutaneous implantation of collagen spheroids

For animal study, three different groups of collagen spheroids (Col, Col/hFDM, Col/hFDM-hep) (*n* = 4, each group) were prepared separately. To make collagen spheroids, hPMSCs suspension were mixed with collagen (3 mg/mL), 50 ng/ml TGF-β1 and hFDM or hFDM-hep together in an ice-bath. Collagen droplets (20 μL) were then pipetted into a 6-well culture dish and cultivated at 37 °C. Six nude mice were anaesthetized and an incision was made at the dorsum to create a subcutaneous pocket. Each sample was transplanted and the skin incisions were closed using 4.0 non-absorbable silk sutures (AILEE). Before transplantation, the hPMSCs were pre-labelled via PKH 26 cell tracking dye and the collagen spheroids were pre-conditioned in the chondrogenic media for 3 days. After 4 weeks of post-implantation, the animals were sacrificed by cervical dislocation and the skin tissues at the implantation sites were harvested. All the animal experiments were approved via the Institutional Animal Care and Use Committee of Korea Institute of Science and Technology (IACUC, 2017-016).

### Histology and immunofluorescence

For histological analysis, all the collagen spheroids were fixed in 4% paraformaldehyde solution for 3 h. The sample were dehydrated with serial concentrations of ethanol (50 to 100%), washed with Histo-Clear II (National Diagnostics, Atlanta), and embedded into paraffin blocks. These samples were sectioned in 5 um thickness, then deparaffinized with Histo-Clear II and ethanol on slide glasses. For Safranin-O staining, the samples were stained with weigert’s iron hematoxylin solution for nuclei staining for 10 min and rinsed, then stained with Fast Green (Sigma, F7528) for 5 min for cytoplasm staining, quickly washed in acetic acid. Finally the slide glasses were stained with Safranin-O solution (Sigma, s8884) for 5 min, dehydrate and cleared with xylene.

For *von Kossa* staining, the slides were rinsed with distilled water and incubated with silver nitrate solution (1%, *w*/*v*) in a clear glass jar under ultraviolet light (60 watt) for 1 h. After washed with distilled water, unreacted silver nitrate was removed with sodium thiosulfate solution (5%, w/v) for 5 min and the samples were counterstained with nuclear fast red (Sigma, N3020) for 5 min. For dehydration, samples were washed and went through graded ethanol and cleared in xylene. Those samples were observed using an optical microscope (Zeiss).

Additionally the collagen spheroids were cryo-sectioned for immunofluorescence of collagen type II. The specimens were washed two times with PBS, blocked for 45 min with 3% BSA. Once they were incubated overnight with a mouse monoclonal anti-Col II (sc-7763; Santa cruze) (1:50) at 4 °C, the samples were washed three times with PBS, incubated for 1 h with Alexa Fluor 488 goat anti-mouse IgG (1:200) at room temperature, and then rinsed with PBS. DAPI staining was also conducted for nucleic detection. The fluorescent signals of PKH26 and Col II were visualized using confocal microscope (Olympus FV1000).

### Statistical analysis

Statistical analysis was performed using the unpaired student t-test. All data represented the mean values and standard errors. Statistical significance was marked as *(*p* < 0.05), **(*p* < 0.01), or ***(*p* < 0.001).

## Results

### Preparation and characterization of matrix-bound heparin

**Fig. 1:**
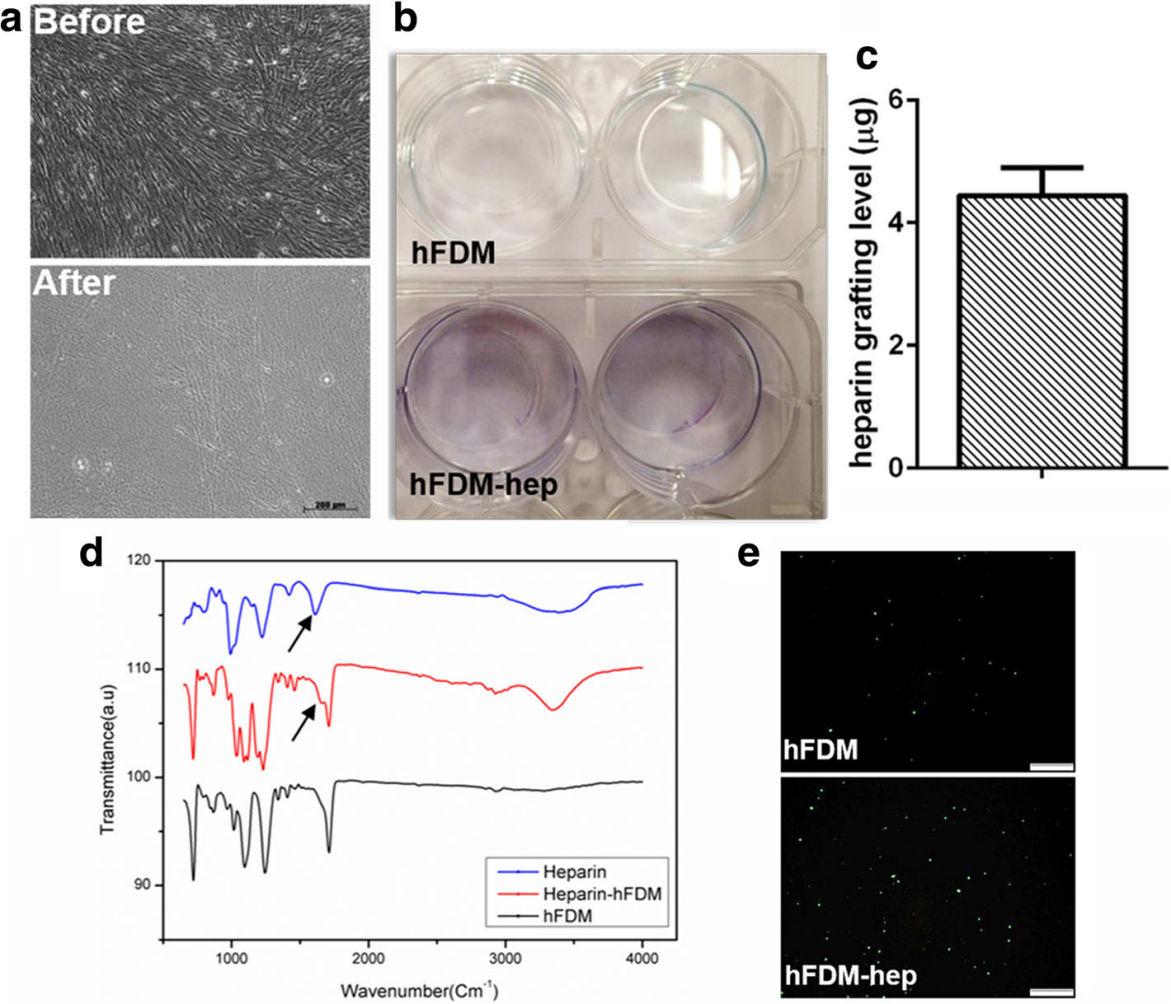
Preparation of hFDM and characterization of heparin-grafted hFDM (hFDM-hep). **a** hFDM morphology before and after decellularization; **b** Toluidine blue staining of hFDM and hFDM-hep; **c** Quantitative measurement of heparin content; **d** Compositional analysis of hFDM-hep using ATR-FTIR; **e** Identification of TGF-β1 immobilized on the hFDM and hFDM-hep, respectively via immunofluorescence of TGF-β1 (green). Scale bar is 200 μm

After the decellularization process was done, we found out that cells completely lost their original morphologies after detergent and enzyme treatments (Fig. 1a). In addition, hematoxylin staining of decellularized ECM was also confirmed via nanofiberous context without the presence of cells (Additional file 1: Figure S1). Upon the application of EDC chemistry, the purple color on hFDM-hep was apparent as indicated by toluidine blue O staining compared to hFDM itself (Fig. 1b). Actual amount of grafted heparin was 4.44 μg upon the initial heparin loading amount of 5 mg (Fig. 1c). ATR-FTIR analysis of heparin and heparin-grafted hFDM exhibited an overlap of sulfate (SO_3_) stretching peaks at 1230 and 1040 cm^− 1^ (Fig. 1d). The results suggest that heparin was successfully conjugated on the hFDM surfaces. In addition, growth factor immobilized on the hFDM or hFDM-hep was visualized via anti-human TGF-β1 antibody. We noticed that positive green spots (bright colors) on hFDM-hep substrate were more pronounced than on hFDM (Fig. 1e).

### Cell viability, proliferation and morphology

**Fig. 2:**
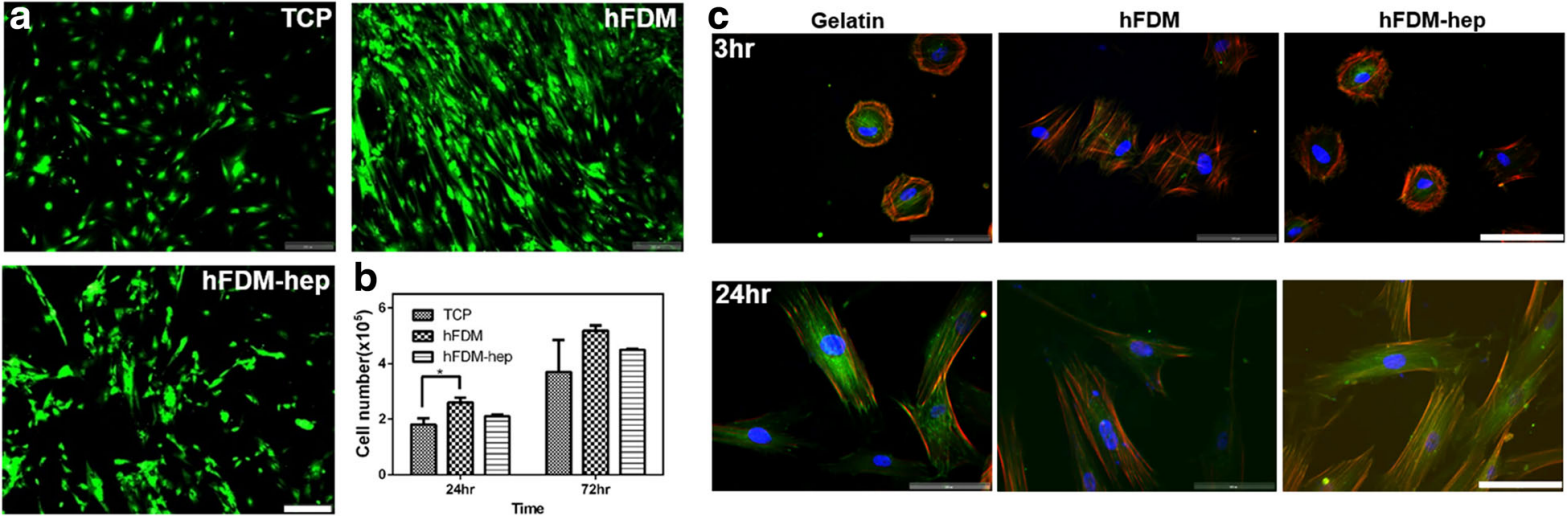
**a** hPMSCs viability on the TCP, hFDM and hFDM-hep using Live/dead staining; **b** Cell proliferation in 24 and 72 h using CCK-8 assay; **c** Observation of cell morphology on different substrates at 3 and 24 h, respectively: DAPI (blue), vinculin (green), and F-actin (red). Scale bar is 100 μm. (*p < 0.05, ** *p* < 0.01, *** *p* < 0.001)

Our results showed that TCP, hFDM and hFDM-hep substrates had similar level of hPMSCs viability, showing little cytotoxicity of each substrate as indicated via live/dead staining after 24 h of cell culture (Fig. 2a). For cell proliferation measurement in 72 h, we found that proliferation rate of hPMSCs was comparable to each other (Fig. 2b). In addition, we observed cell morphologies at 3 and 24 h, respectively (Fig. 2c). However, little difference was found at both time points.

### MSCs encapsulated collagen spheroids and release test of TGF-β1 in vitro

**Fig. 3:**
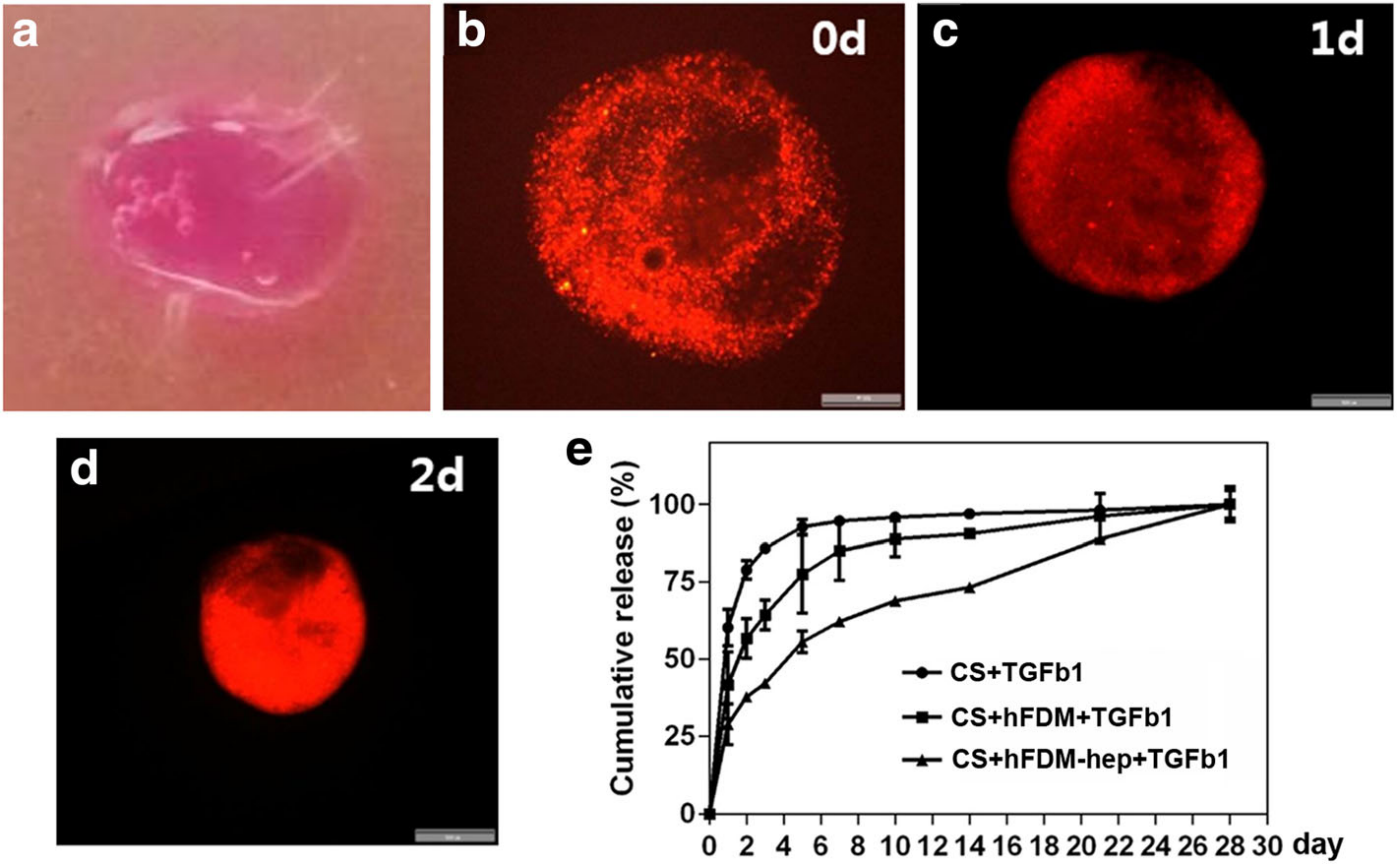
Preparation of collagen spheroids. **a** Collagen spheroids containing hPMSCs and hFDM-hep; **b**–**d** Collagen spheroids containing hPMSCs, hFDM-hep and live cell tracking dye (PKH26) at different time points in vitro; **e** Release profile of TGF-β1 from three different groups of collagen spheroids for 28 days in vitro. CS: collagen spheroid. Scale bar is 200 μm

For hPMSCs encapsulation in 3D environment, collagen spheroids with MSCs and hFDM-hep were prepared (Fig. 3a). They were intended to examine cells viability and their distribution in the spheroids. For this purpose, we labeled hPMSCs with live cell tracking dye (PKH-26, red) and the positive signals of PKH-26 in the spheroids were detectable at different time points (day 0, 1, and 2 day) (Fig. 3b–d). Meanwhile, when the release of TGF-β1 (100 ng) encapsulated in collagen (control), and collagen spheroids containing hFDM or hFDM-hep was examined, respectively individual release profile showed quite different patterns for up to 28 days, especially with a moderate amount of early release for hFDM-hep (Fig. 3e). It suggests that heparin is effective in tethering TGF-β1 and thus in controlling the release rate of TGF-β1.

### Chondrogenic differentiation of hPMSCs in vitro

**Fig. 4:**
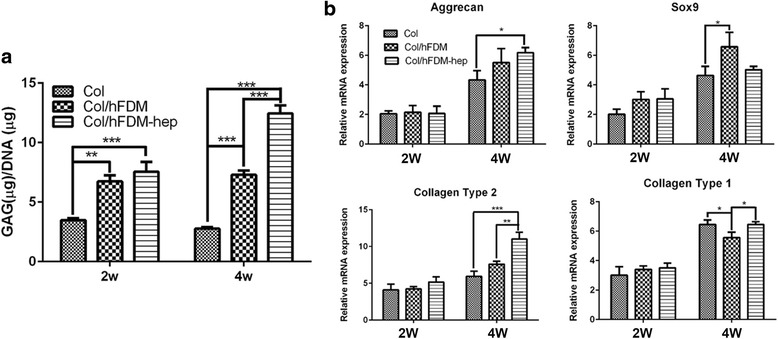
GAG contents and chondrogenic markers expression. **a** The GAG concentrations normalized to DNA content of each sample; **b** Chondrogenic gene expression of positive markers (aggrecan, Sox9, collagen type2) and negative marker (collagen type1) after 2 and 4 weeks of culture in chondrogenic medium. (*p < 0.05, ** *p* < 0.01, ***p < 0.001)

Based on the results of GAG contents measurement, Col alone seems to be not very effective as the GAG concentration stayed low at 2 and 4 weeks (Fig. 4a). Col/hFDM was more effective than collagen group but little GAG increment was found at 4 week. It is notable however that Col/hFDM-hep was the most effective and it showed the highest level of GAG concentration at 4 week. Current results suggested that Col/hFDM-hep may hold an active TGF-β1 and thus lead to advanced chondrogenesis of hPMSCs.

Gene expression level of selected chondrogenic markers shows that at 2 week the expression levels were similar to each other but significant changes were found at 4 week (Fig. 4b). The results unveil that hFDM presented the highest expression of Sox9 and Col/hFDM-hep revealed higher upregulation of aggrecan and collagen type II than the other groups. Collectively, these results suggested that hFDM-hep exerts a very positive effect on the in vitro chondrogenic differentiation of hPMSCs.

### Chondrogenic differentiation of hPMSCs in vivo

**Fig. 5:**
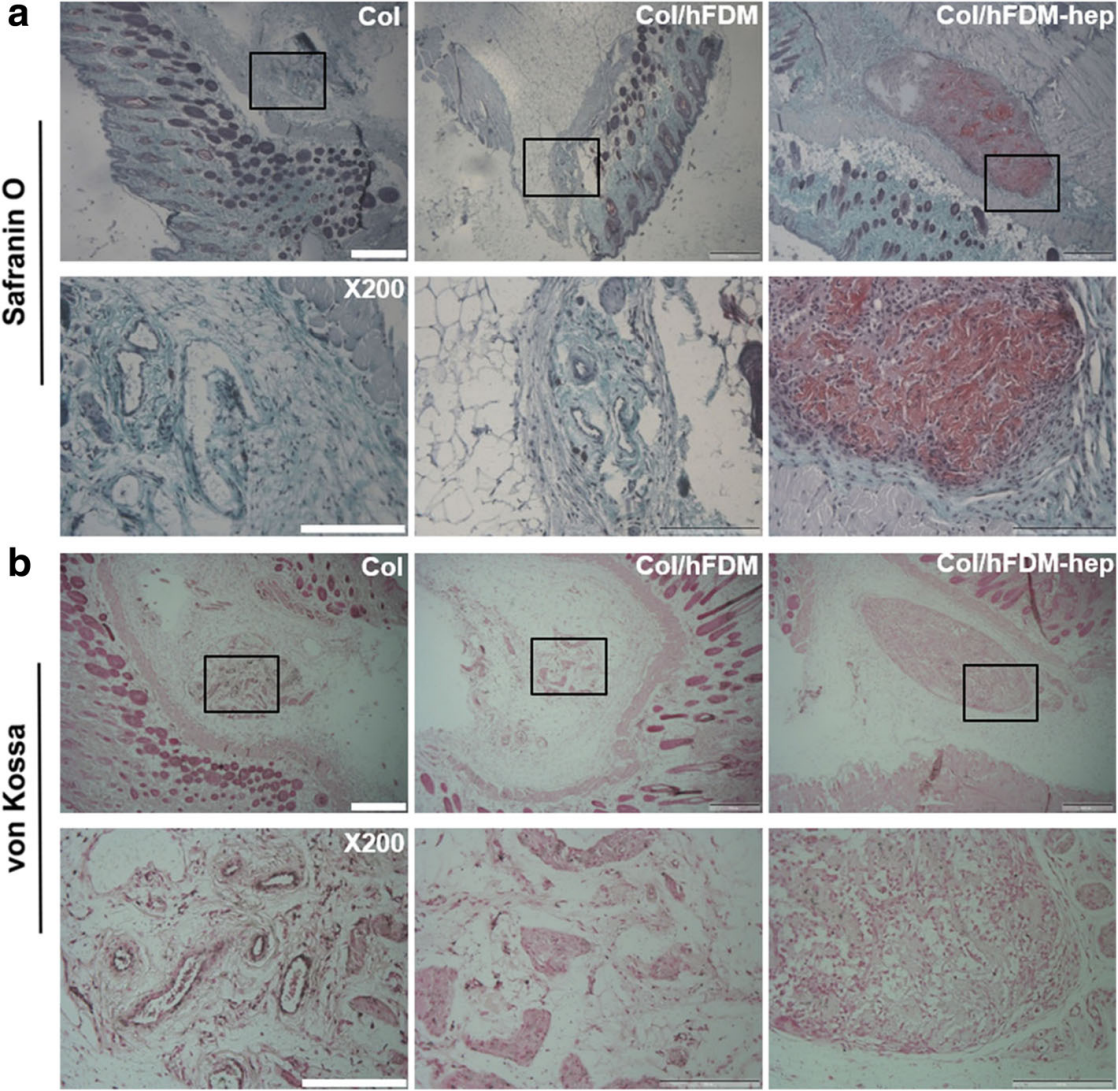
Histological analysis of subcutaneously implanted collagen spheroids at 4 weeks. **a** Safranin-O staining and **b** *von Kossa* staining. The boxed area appears in higher magnification. Scale bar is 500 and 200 μm, respectively

Chondrogenic differentiation of the specimens implanted in vivo was further examined histologically upon retrieved from the implantation sites. According to Safranin-O staining, we found that sulfated GAGs were highly accumulated in Col/hFDM-hep compared to Col and Col/hFDM (Fig. 5a). No significant difference was noticed between Col and Col/hFDM group until 4 weeks. To investigate the chance of osteogenesis differentiation, *von Kossa* staining was performed. The data showed that there was no indication of calcified mineral deposits (no black staining) (Fig. 5b). When the question about whether sulfated GAGs can be derived from hFDM was examined, we saw that sulfated GAGs were barely detectable from hFDM itself via Safranin-o staining (data not shown).

**Fig. 6:**
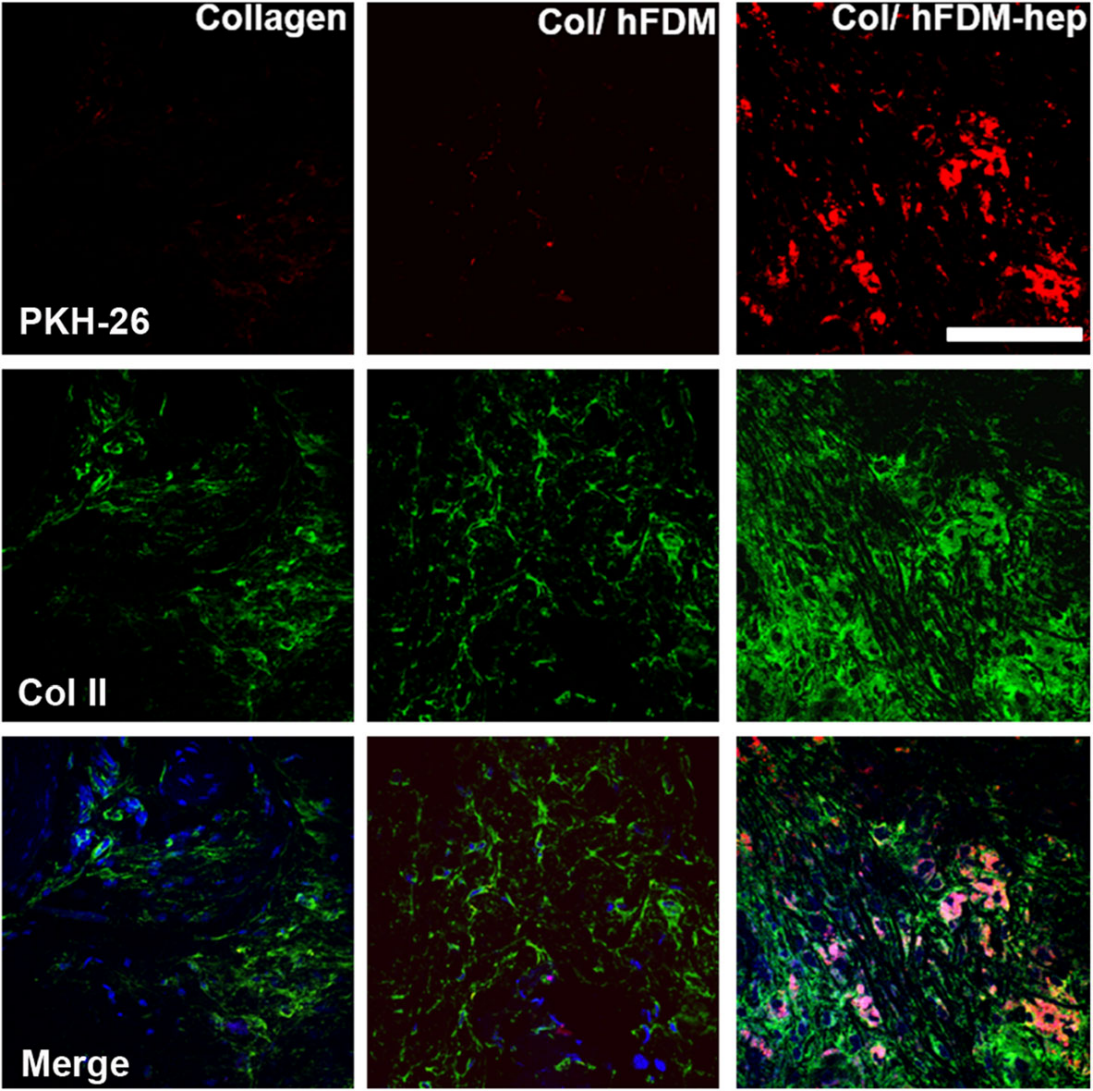
Analysis of chondrogenesis of hPMSCs from subcutaneously implanted collagen spheroids at 4 week. PKH-26 labeled cells (red) and immunofluorescence of Col II (green), and merged images with DAPI staining (blue). Scale bar is 50 μm

Meanwhile as the in vivo specimens were subject to immunofluorescence of collagen type II, the expression of Col II was much stronger with Col/hFDM-hep than Col and Col/hFDM, along with strong PKH-26 positive signals (Fig. 6). These data suggested that there were more chance of viable hPMSCs at 4 weeks in Col/hFDM-hep and that hFDM-hep can provide a suitable microenvironment for chondrogenesis of hPMSCs.

## Discussion

In this study, we investigate induction of hPMSCs for chondrogenesis in 3D collagen spheroid with the support of hFDM-hep and TGF-β1. In our early studies, cell-derived ECM offered favorable microenvironment for MSCs differentiation into osteogenesis and chondrogensis [22, 23]. The development of ideal vehicles for growth factors delivery towards MSCs differentiation has been of great interests. Heparin is well known for its highly sulfated glycosaminoglycan which has a binding affinity to various growth factors such as fibroblast growth factor, vascular endothelial growth factor (VEGF), and TGF-β1 [24]. Previously we reported the use hFDM-hep proved advantageous in delivering an angiogenic growth factor (VEGF) and prompting much better angiognenic activity [25]. In this work, we have tested TGF-β1 immobilized hFDM-hep. TGF-β1 promotes chondrocyte differentiation at early stage by modulating proliferation and increasing alkaline phosphatase activity and proteoglycan synthesis [26, 27]. TGF-β1 on hFDM-hep can be released in a sustained manner for up to 28 days, suggesting that the interaction between heparin and TGF-β1 is secure and effective. The present data demonstrate that current release system of TGF-β1 could be useful in inducing the chondrogenic expression. In addition to TGF-β1 effect on chondrogenesis, the role of heparin itself should be addressed. There have been some reports that incorporation of heparin in the hydrogels can enhance chondrocyte phenotype or re-differentiation of dedifferentiated chondrocytes [28, 29]. The mechanism is reasoned that chondrogenic activity of heparin might be associated with the intrinsic nature of heparin that would secure endogenous growth factors (i.e., TGF-β) secreted from the cells, which can promote the chondrogenesis as a result.

## Conclusions

This study successfully fabricated heparin-grafted hFDM and TGF-β1 tethering onto it. hFDM-hep proves non-toxic and supports the growth of hPMSCs. Encapsulated in collagen spheroids with hPMSCs, hFDM-hep showed an improved chondrogenic potential in vitro and in vivo. This may be due to prolonged bioactivity of TGF-β1, where it was immobilized onto hFDM-hep. Taken together, TGF-β1-immobilized hFDM-hep can provide a beneficial microenvironment for chondrogenesis of MSCs and thus find further applications in cartilage tissue engineering.

## Additional file

Additional file 1: **Figure S1.** Optical image of decellularized hFDM after hematoxylin staining. It shows a nanofiberous ECM structure without the presence of cells. (DOCX 2570 kb)

## References

[1] Martel-Pelletier J, Barr AJ, Cicuttini FM, Conaghan PG, Cooper C, Goldring MB (2016). Osteoarthritis. Nat Rev Dis Primers.

[2] Iwasa J, Engebretsen L, Shima Y, Ochi M (2009). Clinical application of scaffolds for cartilage tissue engineering. Knee Surg Sports Traumatol Arthrosc.

[3] Getgood A, Brooks R, Fortier L, Rushton N (2009). Articular cartilage tissue engineering: today’s research, tomorrow’s practice?. J Bone Joint Surg Br.

[4] Cucchiarini M, Madry H, Guilak F, Saris DB, Stoddart MJ, Koon Wong M (2014). A vision on the future of articular cartilage repair. Eur Cell Mater.

[5] Place ES, Evans ND, Stevens MM (2009). Complexity in biomaterials for tissue engineering. Nat Mater.

[6] Dvir T, Timko BP, Kohane DS, Langer R (2011). Nanotechnological strategies for engineering complex tissues. Nat Nanotechnol.

[7] Huey DJ, Hu JC, Athanasiou KA (2012). Unlike bone, cartilage regeneration remains elusive. Science.

[8] Mano JF, Silva GA, Azevedo HS, Malafaya PB, Sousa RA, Silva SS (2007). Natural origin biodegradable systems in tissue engineering and regenerative medicine: present status and some moving trends. J Royal Soc Interface.

[9] Watt FM, Huck WTS (2013). Role of the extracellular matrix in regulating stem cell fate. Nat Rev Mol Cell Biol.

[10] Frantz C, Stewart KM, Weaver VM (2010). The extracellular matrix at a glance. J Cell Sci.

[11] Hoshiba T, Lu H, Kawazoe N, Chen G (2010). Decellularized matrices for tissue engineering. Expert Opin Biol Ther.

[12] Fitzpatrick LE, McDevitt TC (2015). Cell-derived matrices for tissue engineering and regenerative medicine applications. Biomater Sci.

[13] Jeon J, Lee MS and Yang HS. Differentiated osteoblasts derived decellularized extracellular matrix to promote osteogenic differentiation. Biomater Res. 2018;22:4. 10.1186/s40824-018-0115-0 PMC5824473 29484201

[14] Cen L, Liu W, Cui L, Zhang W, Cao Y (2008). Collagen tissue engineering: development of novel biomaterials and applications. Pediatr Res.

[15] Dhandayuthapani B, Yoshida Y, Maekawa T, Kumar DS (2011). Polymeric scaffolds in tissue engineering application: a review. Inter J Polymer Sci.

[16] Pittenger MF, Mackay AM, Beck SC, Jaiswal RK, Douglas R, Mosca JD (1999). Multilineage potential of adult human mesenchymal stem cells. Science.

[17] Cho H, Lee A and Kim K. The effect of serum types on Chondrogenic differentiation of adipose-derived stem cells. Biomater Res. 2018;22:6. 10.1186/s40824-018-0116-z PMC5845156 29556415

[18] Si YL, Zhao YL, Hao HJ, Fu XB, Han WD (2011). MSCs: biological characteristics, clinical applications and their outstanding concerns. Ageing Res Rev.

[19] Bobis S, Jarocha D, Majka M (2006). Mesenchymal stem cells: characteristics and clinical applications. Folia Histochem Cytobiol.

[20] Vellasamy S, Sandrasaigaran P, Vidyadaran S, George E, Ramasamy R (2012). Isolation and characterisation of mesenchymal stem cells derived from human placenta tissue. World J Stem Cells.

[21] Parolini O, Caruso M (2011). Review: preclinical studies on placenta-derived cells and amniotic membrane: an update. Placenta.

[22] Kim IG, Hwang MP, Du P, Ko J, Ha C, Do SH, Park K (2015). Bioactive cell-derived matrices combined with polymer mesh scaffold for osteogenesis and bone healing. Biomaterials.

[23] Kim IG, Ko J, Lee HR, Do SH, Park K (2016). Mesenchymal cells condensation-inducible mesh scaffolds for cartilage tissue engineering. Biomaterials.

[24] Chung HJ, Kim HK, Yoon JJ, Park TG (2006). Heparin immobilized porous PLGA microspheres for angiogenic growth factor delivery. Pharm Res.

[25] Du P, Hwang MP, Noh YK, Subbiah R, Kim IG, Bae SE, Park K (2014). Fibroblast-derived matrix (FDM) as a novel vascular endothelial growth factor delivery platform. J Control Release.

[26] Zhen G, Cao X (2014). Targeting TGFβ signaling in subchondral bone and articular cartilage homeostasis. Trends Pharmacol Sci.

[27] Grimaud E, Heymann D, Rédini F (2002). Recent advances in TGF-beta effects on chondrocyte metabolism: potential therapeutic roles of TGF-beta in cartilage disorders. Cytokine Growth Factor Rev.

[28] Kim M, Kim SE, Kang SS, Kim YH, Tae G (2011). The use of de-differentiated chondrocytes delivered by a heparin-based hydrogel to regenerate cartilage in partial-thickness defects. Biomaterials.

[29] Brown G, Lim KS, Farrugia BL, Hooper GJ, Woodfield T (2017). Covalent incorporation of heparin improves chondrogenesis in photocurable gelatin-methacryloyl hydrogels. Macromol Biosci.

