# Supplementary material for: Induction of chondrogenesis of human placenta-derived mesenchymal stem cells via heparin-grafted human fibroblast derived matrix
Source: Biomater Res. 2018 May 9;22:12. doi: 10.1186/s40824-018-0121-2 (PMC5941790; doi:10.1186/s40824-018-0121-2)
Supplement: Supplementary file 1 — Figure S1. Optical image of decellularized hFDM after hematoxylin staining. It shows a nanofiberous ECM structure without the presence of cells. (DOCX 2570 kb) [file 40824_2018_121_MOESM1_ESM.docx]

**Supplementary Information**

**Induction of chondrogenesis of human placenta-derived mesenchymal stem cells via heparin-grafted human fibroblast derived matrix**

Yong Kwan Noh^1,2^, Ping Du^1^, Avelino Dos Santos Da Costa^1,3^ and Kwideok Park^1,3 *^

^1^Center for Biomaterials, Korea Institute of Science and Technology, Seoul 02792, Korea

^2^Dept of Biotechnology, Korea University, Seoul 02841, Korea

^3^Division of Bio-Medical Science and Technology, KIST School, Korea University of Science and Technology (UST), Seoul 02792, Korea

Running title: Induction of chondrogenesis of hPMSCs

Submitted to *Biomaterials Research*

*Correspondence: Kwideok Park, Ph.D.

E-mail: kpark@kist.re.kr

Tel: +82-2-958-5288

Fax: +82-2-958-5308

January 2018


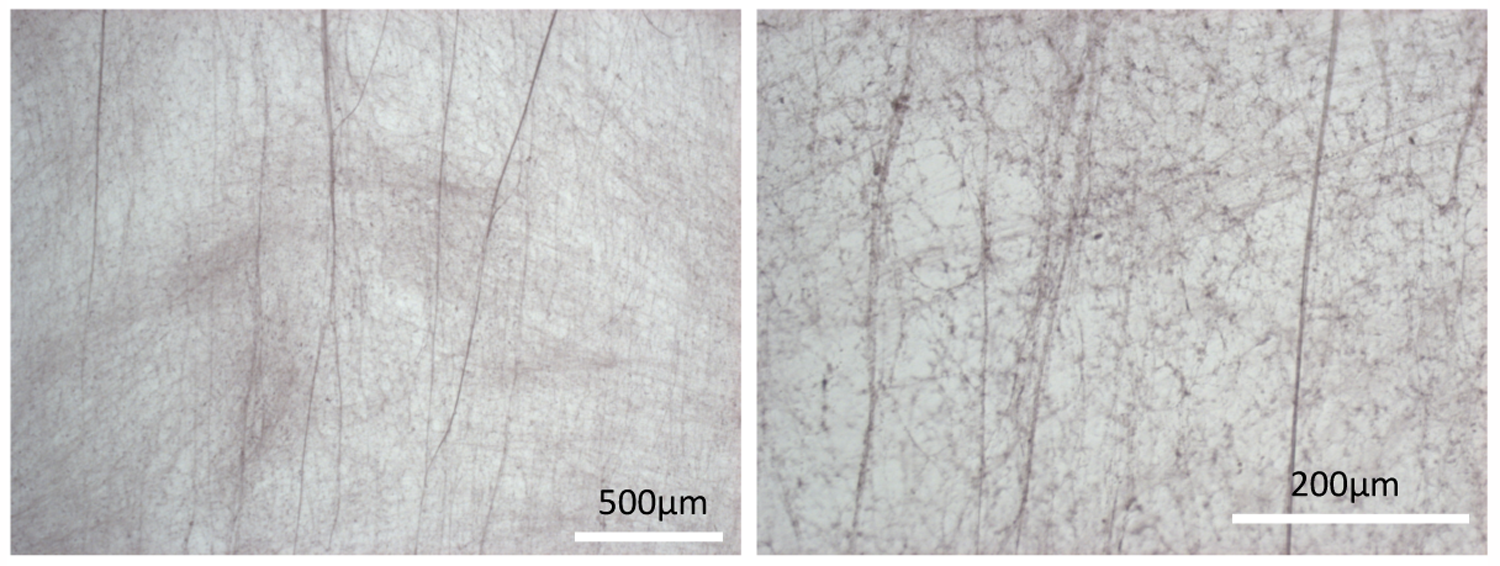


**Fig. S1.** Optical image of decellularized hFDM after hematoxylin staining. It shows a nanofiberous ECM structure without the presence of cells.
